# Analysis of the prevalence and incidence of pseudohypoparathyroidism in Poland based on National Health Fund data with clinical presentation of own cases

**DOI:** 10.3389/fendo.2025.1584154

**Published:** 2025-05-15

**Authors:** Arkadiusz Zygmunt, Monika Pacocha, Agnieszka Domanska-Czajka, Elzbieta Skowronska-Jozwiak, Anna Lupinska, Renata Stawerska, Katarzyna Wojciechowska-Durczynska, Agnieszka Gach, Malgorzata Karbownik-Lewinska, Andrzej Lewinski

**Affiliations:** ^1^ Department of Endocrinology and Metabolic Diseases, Medical University of Lodz, Lodz, Poland; ^2^ Department of Endocrinology and Metabolic Diseases, Polish Mother’s Memorial Hospital - Research Institute, Lodz, Poland; ^3^ Department of Pediatric Endocrinology, Medical University of Lodz, Lodz, Poland; ^4^ Department of Genetics, Polish Mother’s Memorial Hospital - Research Institute, Lodz, Poland; ^5^ Polish Mother’s Memorial Hospital – Research Institute, Lodz, Poland

**Keywords:** pseudohypoparathyroidism (PHP), prevalence, incidence, Albright hereditary osteodystrophy (AHO), hypocalcemia

## Abstract

**Introduction:**

Pseudohypoparathyroidism (PHP) is a rare genetically determined disease with a wide range of symptoms related to target organs’ resistance to parathyroid hormone (PTH). Lack or insufficient action of PTH on effector organs causes hypocalcemia and hyperphosphatemia. Some patients may have characteristic features of Albright hereditary osteodystrophy (AHO).

**Methods:**

We estimated the prevalence and incidence of PHP using National Health Fund (NHF) data between 2013 and 2023.

**Results:**

The prevalence was 0.62-0.64/100,000 person-years for Poland and 1.11-1.47/100,000 person-years for the Lodz Voivodeship, depending on the analysis period, i.e., between 2013 and 2019, 2020 and 2021, and 2022 and Oct 2023. During these periods in Poland, PHP was diagnosed for the first time in 282, 32, and 29 patients, respectively, which means that the incidence was 0.19/1,000,000, 0.48/1,000,000, and 0.46/1,000,000 person-years, respectively. In all periods, there were more females in the adult group than in the pediatric group. The study also analyzed data on 19 patients with PHP who have been followed at the Polish Mother’s Memorial Hospital – Research Institute (PMMH-RI), describing the clinical presentation of the disease, treatment, and difficulties in establishing the proper diagnosis.

**Discussion:**

In order to quickly establish the correct diagnosis, it is crucial to collect detailed medical interviews with a family history of the disease. It is also essential to carefully perform a physical examination, looking for non-specific symptoms. It’s also important to check a broader panel of biochemical tests at the beginning of the diagnosis, characterizing calcium-phosphate homeostasis rather than just the concentration of total calcium in serum.

## Introduction

Pseudohypoparathyroidism (PHP) is a rare genetically determined disease with a wide range of symptoms related to target organs’ resistance to parathyroid hormone (PTH) (1, 2). The manifestation of the disease is hypocalcemia and hyperphosphatemia of varying severity, despite inadequately elevated levels of PTH. In some individuals, characteristic phenotypic features known as Albright hereditary osteodystrophy (AHO) are observed, including abnormal bone development (i.e., short stature, shortening of the fourth and fifth metacarpal bones —brachydactyly type E), stocky builds, round face, early-onset obesity, and ectopic, often subcutaneous ossifications (3). The clinical presentation usually includes symptoms of intellectual disability and neurocognitive disorders (4, 5). In some cases, resistance to other hormones like thyroid-stimulating hormone (TSH), gonadotropins, growth hormone-releasing hormone (GHRH), and calcitonin is found (6). Due to heterogeneous and not distinctive clinical manifestations, the diagnosis of PHP is typically delayed, leading to postponed therapeutic decisions and increased morbidity resulting from the consequences of untreated disease. There is limited data on the prevalence of PHP. Data from Japan (7), Denmark (8), and Norway (9) indicate prevalence rates were 1.2/100,000, 1.1/100,000, and 0.82/100,000, respectively. The differences may result, among others, from survey methodology (i.e., nationwide mail-based survey (7), analysis of the National Hospital Patient Registry - NPR (8) and electronic hospital registry (9)); however, other factors that are not entirely known may also be significant. Pathogenesis involves variants in the GNAS1 gene or other genes encoding the signaling pathways from the PTH receptors type 1 PTH/PTHrP (PTHR1) (2, 10). PHP is divided into different types: PHP type 1A - PHP1A (Online Mendelian Inheritance in Man - OMIM - #103580), PHP type 1B - PHP1B (OMIM - #603233), PHP type 1C - PHP1C (OMIM - #612462) and PHP type 2 - PHP2 (OMIM - #203330).

In PHP1A, the variant mainly affects the GNAS1 gene inherited from the mother, leading to abnormal signal transduction in many tissues. Patients have a full-blown disease with the AHO phenotype. In other cases, when a gene is inherited from the father, only the AHO phenotype occurs without hormonal and electrolyte disturbances, known as pseudopseudohypoparathyroidism (PPHP) (OMIM - #612463). The AHO phenotype, without multi-hormone resistance, is also observed in progressive osseous heteroplasia (POH) (OMIM - #166350) or osteoma cutis. Patients with preserved bone response to PTH may experience osteitis fibrosa cystica.

Patients with PHP1A due to GNAS1 mutation have reduced activity of the α subunit of the stimulatory G protein — Gsα. Despite, impairment of the signaling pathway for PTH, it leads to resistance to other Gs protein coupled hormones [e.g. thyroid-stimulating hormone (TSH), luteinizing hormone (LH), follicle-stimulating hormone (FSH), growth hormone-releasing hormone (GHRH)] due to paternal imprinting of Gsα transcripts in specific tissues (11).

In PHP1B, the variant usually affects the GNAS1 gene inherited from the mother. Only the kidneys are primarily affected, and the AHO phenotype is absent.

Both PHP1A and PHP1C present the same clinical features, and there are doubts about whether these subtypes should be considered separate entities. The difference is measurement of the Gsa protein activity from various cell types i.e. erythrocyte membranes, patients with PHP1A have a partial deficiency of activity (~50%), however this defect is absent in patients with PHP1C (3).

The exact molecular cause of PHP2 is still unknown, but it probably involves variants affecting downstream signaling pathway from the receptor. Most commonly, it leads to elevation of PTH, hypocalcemia, and decreased bone mineral density (BMD). Typically, there is no resistance to other hormones. The patient phenotype can be similar to PHP1A and PHP1C.

Acrodysostosis (OMIM - #101800) refers to a group of rare skeletal dysplasias that present features typical of PHP, such as resistance to PTH, TSH, and severe brachydactyly. However, unlike PHP, more extensive facial dysmorphia is observed, nasal hypoplasia, and often neurocognitive impairment (12, 13).

The European Network for the study of PHP (EuroPHPnetwork) in 2016 suggested using new nomenclature inactivating PTH/PTHrP signaling disorder (iPPSD) instead of e.g. PHP and PPHP, POH, Acrodysostosis to refer to common pathogenesis of impairments in PTH and/or PTHrP cAMP-mediated pathway (14).

PTH classically affects three organs: the kidneys, bones, and—indirectly—the digestive tract. In PTH resistance, the hormone has no or reduced physiological effect on target cells.

In the renal tubules, calcium reabsorption and excretion of phosphates are inhibited, leading to hypocalcemia and hyperphosphatemia. Hydroxylation of vitamin D at C1, stimulated by PTH, is also reduced, limiting calcium and phosphorus absorption from the gastrointestinal tract.

The extent to which PTH signaling in bone is defective in PHP patients is less known. There is often increased bone resorption, but the clinical presentation may be variable, ranging from decreased to increased BMD, i.e., osteitis fibrosa cystica vs osteosclerosis, respectively (1).

Laboratory indicators of PHP include hypocalcemia, hyperphosphatemia, and elevated or inappropriately high PTH concentration. Hypocalcemia predisposes to increased cell excitability, which translates into frequently occurring sensory symptoms (tingling, numbness) and/or motor symptoms (tetany) (14). Long-term biochemical disorders may lead to ectopic calcifications, including intracerebral and subcutaneous calcifications and enamel defects. Additionally, characteristic but rare ectopic ossifications are observed, not resulting from disturbances in calcium-phosphate homeostasis but from molecular changes in the GNAS gene (15, 16).

PHP treatment aims to reduce clinical symptoms, correct biochemical disorders, and limit the long-term consequences of the disease. For this purpose, hydroxylated metabolites of vitamin D and calcium preparations are used. In the case of concurrent hypothyroidism, L-thyroxine administration is recommended.

## Material and methods

National health insurance system in Poland aims to provide health care for insured people and persons entitled to health care services financed from public funds (including everyone under 18). The National Health Fund (NHF) finances the health services from compulsory health insurance contributions and collects data on the services provided. Therefore, all NHF data concerns hospitalizations and outpatient visits carried out as part of public health care, and they do not include data from private facilities.

Prevalence and incidence were assessed based on NHF data from 01 Jan 2013 to 31 Oct 2023 (17, 18). The data obtained for the analysis was divided into three periods: from 01 Jan 2013 to 31 Dec 2019, i.e., the period before the COVID-19 pandemic; from 01 Jan 2020 to 31 Dec 2021, i.e., the COVID-19 pandemic period; and from 01 Jan 2022 to 31 Oct 2023, the current period.

Prevalence and incidence were estimated based on the number of medical consultations provided (medical services in outpatient and inpatient conditions) and when the primary diagnosis was PHP—E20.1 (according to the International Classification of Diseases 10, ICD-10). It was assumed that the incidence was a sum of reported diagnoses of E20.1 in a given year, the lack of reported diagnoses of E20.1 in the three previous years, and the prevalence was a sum of at least one reported diagnosis of E20.1 during a given year or in the last three years, assuming that the patient was alive at the end of the year. The analysis included age (adults vs. children) and gender. It was also assumed that children are people who, according to their birth year, are less than 18 years old, and adults are people aged 18 or older. The method of analyzing the results was arbitrarily adopted by the NHF. The prevalence and incidence were estimated for Poland and Lodz Voivodeship, comparing the NHF data with the patients’ register at the PMMH-RI. The sensitivity threshold was 5 events in a given subgroup. This criterion was arbitrarily adopted by the NHF and could not be changed by us for procedural reasons. In some cases, it was possible to clarify the source data. The obtained data do not specify how many patients had a disease diagnosis confirmed by genetic testing. The medical data of 55 patients treated at the PMMH-RI in the years 1998–2023 diagnosed with PHP were also analyzed. The retrospective analysis concerned both hospitalized and outpatient patients. The diagnosis of PHP was made based on the analysis of biochemical tests and the clinical presentation. Due to the lack of medical data, only 19 patients (11 women/girls and 8 men/boys) have been subjected to the final analysis. Patients were aged from 1 to 61 years (mean age ± standard deviation - 32.9 ± 19.4 years). Anthropometric measurements (height, body mass), signs and symptoms of the disease, the period from the appearance of clinical symptoms to diagnosis, and the age at diagnosis were analyzed. Additionally, the results of laboratory tests (PTH, total calcium, inorganic phosphorus in serum) before and after pharmacological treatment, imaging tests (X-ray of the hand, CT scan of the head), and genetic MS-MPLA tests were analyzed. The SALSA MS-MLPA Probemix ME031 GNAS (MRC Holland) is an assay enabling detection of aberrant methylation of several sequences of the GNAS complex locus. Moreover, the probemix also allows to detect deletions/duplications in the GNAS complex locus and the STX16 gene.

The analysis of pharmacological treatment included the doses of drugs the patient used most often.

### Statistical analysis

The data were statistically analyzed using non-parametric test (Mann-Whitney Rank Sum test), and Chi-Square analysis. In all analyses, statistical significance was accepted at p < 0.05. Data processing, statistical analyses, and figures were performed by using SigmaPlot 12.3 (Systat Software, Inc., San Jose, CA, USA) and Excel (Microsoft Corp., Redmond, WA, USA).

The Ethics Committee at PMMH-RI approved the protocol (KB/66/2023).

## Results

In Poland from January 2022 to October 2023, PHP was treated in 388 patients, corresponding to a disease prevalence rate of 0.62/100,000 person-years. Among them, 251 were adults (162 females and 89 males), and 137 are children (64 girls and 73 boys), which indicates that the frequency of PHP occurrence among women in the adult group is higher compared to the children group (64.5% vs 35.5% and 46.7% vs 53.3%, respectively, p<0.001).

From 2013 to 2019 and 2020 to 2021, patients with PHP were 1481 and 431, respectively, indicating a similar prevalence rate of 0.62/100,000 and 0.63/100,000 person-years, respectively.

In both periods, there are more females in the adult than in the pediatric group, in 2013-2019: 67.4% vs. 32.6% and 51.9% vs. 48.1%, respectively (p<0.001); and in 2020-2021: 66.6% vs. 33.4% and 52.1% vs. 47.9%, respectively (p=0.008). The data on number of treated patients and prevalence rate in Poland and Lodz Voivodeship are summarized in **Table 1**.

**Table 1:** Number of patients with PHP and prevalence of the disease in Poland and Lodz Voivodeship.

Period	Poland							Lodz Voivodeship						
	Numbers of patients					Insured persons (10,000)	Prevalence (per 10,000 person-years)	Numbers of patients					Insured persons (10,000)	Prevalence (per 10,000 person-years)
	Children		Adult		All			Children		Adult		All		
	F	M	F	M				F	M	F	M			
2013-2019	194	180	746*	361	1481	338.34	0.63	27	26	81	50	184	23.673	1.11
2020-2021	61	56	209**	105	431	336.27	0.64	5	11	29	21	66	22.377	1.47
2022 - Oct-2023	64	73	162*	89	388	341.28	0.62	4	11	21	17	53	22.211	1.30

* The difference in the frequency of the disease occurrence among women in the adult group compared to children (p<0.001).

** The difference in the frequency of the disease occurrence among women in the adult group compared to children (p=0.008).

Oct, October; F, female; M, male.

From Jan 2022 to Oct 2023 in Lodz Voivodeship, 53 patients with PHP health service were provided, which indicates a prevalence rate of 1.31/100,000 person-years. In previous periods (2013–2019 and 2020-2021), the prevalence rate was 1.11/100,000 and 1.47/100,000 person-years, respectively.

Due to the established sensitivity threshold at 5 events by the NHF, and the small number of PHP cases, the prevalence and incidence rates were not specified with the division into gender and between children and adults.

**Figure 1:**
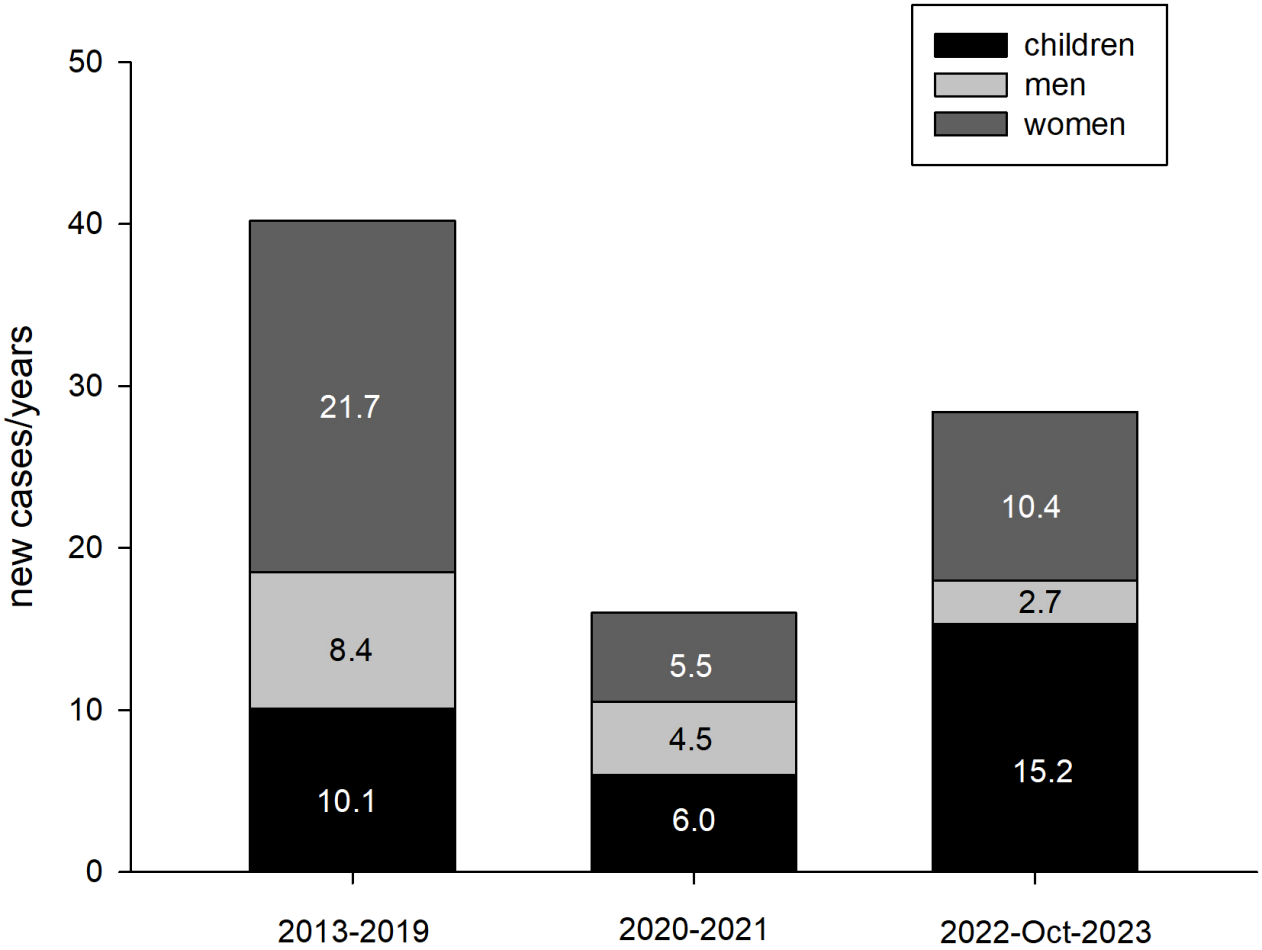
The number of new cases of PHP in 3 examined periods, taking into account their duration (person/years).

Between 2022 and Oct 2023 in Poland, PHP was diagnosed for the first time in 29 patients (11 girls, 17 boys, 19 females, and 5 males). Between 2013 and 2019, PHP was diagnosed in 282 patients (28 girls, 43 boys, 152 females, and 59 males). Between 2020 and 2021, there were 32 patients (5 girls, 7 boys, 11 females, and 9 males). The incidence rate was 1.19/1,000,000 (2013 to 2019); 0.48/1,000,000 person-years (2020-2021), and 0.46/1,000,000 person-years (2022 to Oct 2023) (p<0.001). The number of new cases with PHP in the following periods, taking into account their duration, is presented in **Figure 1**.

**Figure 2:**
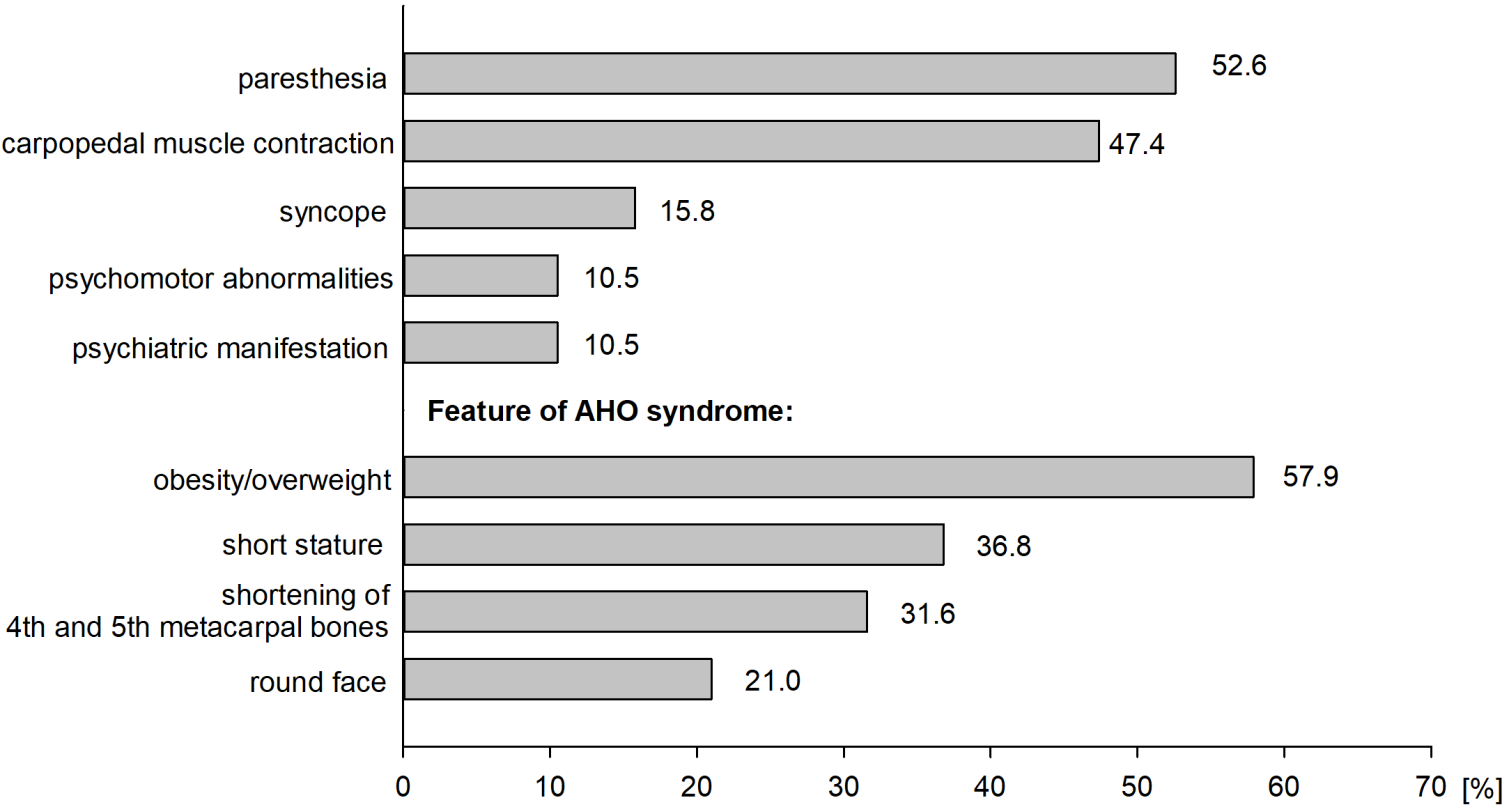
Clinical manifestation of patient with PHP.

The study analyzed data on 19 patients with PHP who have been followed at the PMMH-RI. Among them, 11 are females (57.9%) and 8 are males (42.1%). The characteristics of the analyzed group are presented in **Table 2**. The average age for diagnosis of PHP was 20.4 ± 17.8 years. The median duration from the onset of symptoms to the final diagnosis was 37.8 months. The most frequently reported symptoms were obesity/overweight (57.9%) and paresthesia (52.6%). Clinical manifestation of patients with PHP is presented in **Figure 2**. The most extended period from the onset of symptoms to the diagnosis was recorded among patients with symptoms of hypocalcemia - muscle contraction and paresthesias (53.1 ± 63.9 months). The shortest period was observed among patients without the abovementioned symptoms despite its associated hypocalcemia (8.0 ± 3.5 months). However, most of this group consists of patients in whom the disease was diagnosed in a family member shortly before their diagnosis. The duration from the appearance of signs and symptoms to the appropriate diagnosis in patients with a positive or negative family history of PHP was compared (8.2 vs 52.1 months, respectively, non-significant). The first case in the family was considered as a patient without a family history of disease. Among 12 patients (75.0%), PHP was diagnosed in at least one family member, the remaining cases were assessed as sporadic (25.0%).

**Table 2:** Characteristics of patients with PHP treated at PMMH-R.

Parameter	n (%)	Value average ± SD(min-max)	median(1 and 3 quartile)
Gender	19		
Female	11 (57.9%)		
Male	8 (42.1%)		
Age, years	19	32.9 ± 19.4 (1-74)	28.5 (20.5; 50)
Female	11 (57.9%)	34.8 ± 17.8 (10-61)	32 (20.5; 50)
Male	8 (42.1%)	30.4 ± 22.4 (1-74)	25 (20; 34.5)
Height, cm - Adults	13		
Female	7	153 ± 7,3 (145-164)	152 (147; 158.5)
Male	6	172 ± 7.8 (156-177)	174 (173; 175.7)
Not available	2	N/A	N/A
BMI, kg/m^2^ – Adults	13	24.7 ± 4.5 (17.6-34.2)	23.7 (22.6; 26.8)
Female	7	25.5 ± 6.0 (17.6-34.2)	26.8 (21.05; 29.1)
Male	6	23.7 ± 1.9 (21.0-26.1)	23.3 (22.7; 25.2)
Not available	2	N/A	N/A
BMI, kg/m^2^ – Children	4	29.2 ± 4.9 (25.4-36.3)	27.5 (26.6; 30.1)
Age at time of diagnosis, years	19	20.4 ± 17.8 (0.1-58)	15 (8; 25.5)
patients with hypocalcemia symptoms (muscle contraction, paresthesias)	10	23.2 ± 16.5 (0.1-53)	20 (14.2; 25.7)
patients without hypocalcemia symptoms	5	15.8 ± 15.4 (1-58)	13 (6; 15)
patients with normocalcemia	3	6.0 ± 5.6 (1-12)	5 (3; 8.5)
patients with positive family history of PHP (before diagnosis)	8	20.9 ± 19.7 (3-58)	14 (5.7; 29.2)
patients with a negative family history of PHP	11	20.0 ± 17.2 (0.1-53)	15 (11; 23.5)
Period from the onset of symptoms to the diagnosis of PHP, months	15	37.8 ± 54.7 (1-168)	9 (3.25; 51)
patients with hypocalcemia symptoms (muscle contraction, paresthesias)	9	53.1 ± 63.9 (1-168)	12 (3; 108)
patients without hypocalcemia symptoms	3	8.0 ± 3.5 (6-12)	6 (6; 9)
patients with normocalcemia	3	17.0 ± 12.1 (3-24)	24.0 (13.5; 24)
patients with a positive family history of PHP (before diagnosis)	5	8.2 ± 9.0 (2-24)	6 (3; 6)
patients with a negative family history of PHP	10	51.2 ± 60.1 (1-168)	18 (6; 96)
Not available	4	N/A	N/A
At least one family member with diagnosed PHP (n=16)	12 (75.0%)		
Presence of GNAS mutation in genetic test (n=13)	8 (61.5%) PHP1A – 2 PHP1B – 6		
Presence of cerebral calcifications in the head - CT (n=10)	8 (80.0%)		
Presence of shortening of the fourth and fifth metacarpal bones in hand - X-Ray (n=12)	6 (50%)		
Treatment of hypothyroidism (n=19)	16 (84.2%)		

n, number; min, minimum; max, maximum; BMI, body mass index; CT, computed tomography; PHP, pseudohypoparathyroidism; SD, standard deviation, N/A, not available.

Genetic testing was performed in 13 patients. Eight patients (61.5%) were found to harbor pathogenic GNAS variant, or intragenic STX16 deletion, among whom PHP1A and PHP1B were confirmed in 2 and 6 patients, respectively. During the performed diagnostics, 10 patients had head CT, and 8 patients (80.0%) cerebral calcifications were detected. Additionally, 6 patients had shortening of the fourth and fifth metacarpal bones in hand X-ray. Sixteen patients (84.2%) were treated for hypothyroidism.

The retrospective analysis included biochemical parameters of calcium-phosphate metabolism (PTH, calcium total, inorganic phosphorus). The values of the results before and during the treatment with active vitamin D metabolites and calcium supplements are presented in **Table 3**. Normocalcemia was observed among 3 patients (15.8%). In the treatment of patients with PHP, active metabolites of vitamin D, calcium supplements, and cholecalciferol were used. A majority of patients were treated with alfacalcidol (73.4%). The presentation of averaged doses of medications used by the patients is shown in **Table 4**.

**Table 3:** Biochemical features of patients with PHP (n=13).

Parameter	normal range	Before treatment		After treatment	
		average ± SD (min-max)	median (1 and 3 quartile)	average ± SD (min-max)	median (1 and 3 quartile)
PTH n=13	15–65 pg/mL	305.2 ± 218.9 (39.3-735)	223.2 (151; 447.2)	178.8 ± 168.0 (40-620.9)	102.2 (69.9; 206.0)
Calcium total n=13	2.2-2.65 mmol/L	1.8 ± 0.5 (0.9-2.6)	1.8 (1.4; 2.2)	2.2 ± 0.2* (1.9-2.6)	2.3 (2.1; 2.4)
Phosphates n=13	0.81-1.45 mmol/L	1.9 ± 0.4 (1.3-2.7)	2.0 (1.7; 2.1)	1.5 ± 0.3 (1.2-2.0)	1.5 (1.3; 1.9)
Calcium phosphate product n=13	<55 mg^2^/dL^2^	43.0 ± 12.1 (25.0-62.5)	43.6 (31.1; 54.2)	42.5 ± 9.1 (30.0-59.5)	41.9 (34.8; 48.6)
Not available (n=6)		N/A	N/A	N/A	N/A

* Difference between serum calcium concentration after treatment vs. before treatment was statistically significant (p=0.002).

PTH, parathormone; SD, standard deviation, N/A, not available; n, number.

**Table 4:** Drugs and doses used in treating patients with PHP (n=16).

Drug	n (%)	average ± SD (min-max)	median (1 and 3 quartile)
Alfacalcidol, µg/d	14 (87.5%)	1.5 ± 0.9 (0.25-3)	1.25 (1.0; 2.1)
Calcitriol, µg/d	2 (12.5%)	0.4 ± 0.2 (0.25-0.5)	0.4 (0.3; 0.4)
Calcium carbonate, mg/d	14 (87.5%)	1642 ± 1008.2 (500-3000)	1250 (1000; 2750)
Cholecalciferol, IU/d	14 (87.5%)	1530.8 ± 1097.3 (400-4000)	1000 (1000; 2000)
L-thyroxine, µg/d	16 (100%)	68.2 ± 25.2 (25-112)	75 (59.3; 81.5)

SD, standard deviation.

## Discussion

### Prevalence and incidence

Based on the presented data, it can be concluded that the prevalence of PHP in Poland was very similar in the analyzed periods and was in the range of 0.62-0.64/100,000 and it seems to be lower compared to PHP prevalence in other countries where it ranged from 0.82-1.2 per 100,000 inhabitants (7–9). This value is influenced by many factors, including the quality of a given country’s healthcare system and how data is obtained. An example here is two studies from Japan, which determined the prevalence of PHP in 1997 at 0.34/100,000 (19) and in 2017 at 1.2/100,000 (7). Although both analyses were based on surveys, the research details were different. In the first research work, the questionnaire was sent randomly to various medical entities (19), and in the second - it was addressed to the largest hospitals, assuming that they mainly focus on patients with PHP (7). This diversity of research techniques can explain the differences between the estimated prevalence for Poland and other countries (7–9) and the differences between the estimated prevalence for the Lodz Voivodeship, which is higher than the estimated prevalence for Poland. Due to its central location and the number of inhabitants, Lodz has many medical entities with the highest level of reference, and the Department of Endocrinology and Metabolic Diseases of the PMMH-RI is a reference center for the treatment of rare, endocrine and metabolic diseases, both in children and adults. If the 4-fold increase in the prevalence of PHP over a 20-year period, noticed by the Japanese authors, was determined only by the development of the healthcare system, our 10-year observations should also capture these differences, but none were observed. In addition, changes in the functioning of health care during the COVID-19 pandemic should also affect prevalence, which was not noticed in our studies. However, over the years, the incidence decreased (from 1.19/1,000,000 person-years for the period before the COVID-19 pandemic and 0.46-0.48 per 1,000,000 person-years since the beginning of the COVID-19 pandemic), what may express the limited availability of patients to the healthcare system during the COVID-19 pandemic. The distribution of recognized new PHP cases has also changed. There was a greater increase in new diagnoses of PHP in children (15.2 person-years from 2022 to Oct 2023 vs. 10.1 person-years from 2013 to 2019 and 6 person-years from 2020 to 2021; p<0.001), which may reflect a more effective diagnosis of the disease. The most important factor influencing the diagnosis of new cases of PHP is that it is a disease with a complex and diverse pathogenesis, which determines the heterogeneous clinical picture.

### Diagnostic difficulties

In the analyzed group, the first clinical presentation most often reported were symptoms of hypocalcemia, i.e., paresthesia and muscle spasms. Despite these symptoms, diagnosis took an average of 53 months, and the duration was extensive and ranged from 1 to 168 months. At that time, patients were most often diagnosed with, among others, epilepsy and mental disorders. Therefore, in the case of clinical symptoms of hypocalcemia, especially mild ones, it is essential to determine the concentration of calcium in the blood serum, corrected for the concentration of albumin, but also other parameters of the calcium-phosphate metabolism, such as the concentration of phosphates, PTH, magnesium and 25(OH)D and monitoring of kidney function. The first biochemical manifestation in patients with PHP is usually an increased PTH concentration, then an increase in the concentration of inorganic phosphorus in the serum, and finally, the development of hypocalcemia (20), so the finding of a normal calcium concentration in the serum does not exclude the disease. Moreover, hypocalcemia does not always have to be symptomatic. In 3 patients with hypocalcemia, no clinical symptoms were observed. These people were tested because the disease occurred in a close family member. In the presented analysis, a positive family history of PHP shortened the diagnostic process several times (8.2 vs. 51.2 months).

Among the patients of the PMMH-RI, there are 9 families suffering from PHP. In one of them, the diagnosis of PHP1B confirmed by genetic testing was made in 5 people. All affected family members showed a complete lack of methylation in exon A/B of the GNAS gene corresponding to an intragenic deletion of the STX16 gene involving exons 5 and 6.

The history of the family disease began in 1975 with an unexplained loss of consciousness accompanied by muscle spasms in a 12-year-old girl. Epilepsy was then suspected, but 14 years later (1989), a full-blown tetany attack occurred, and the diagnosis was verified. After a few years, PHP was confirmed by molecular tests. After symptoms of the disease appeared in the patient’s two daughters (tingling in the hands, muscle spasms), sister (depressive episode), and nephew (mental disorders), the diagnosis lasted 2, 3, 6, and 6 months, respectively. The story of this family perfectly demonstrates the importance of conducting a medical interview regarding the family history of genetic diseases. This may be crucial when choosing the right direction of the diagnostic path, resulting in faster diagnosis of the disease and satisfactory treatment. In patients carrying the exact genetic change, the clinical presentation and severity of the disease vary greatly. Each of the above-described patients required different, individually selected doses of drugs (alfacalcidol from 1 to 3 µg/day and calcium carbonate from 500 to 3000 mg/day).

There are also no clear criteria for diagnosing AHO, i.e., which signs and phenotypic features of AHO should be present. Criteria/clinical symptoms are divided into basic (brachydactyly type E and short stature) and additional (stocky build, round face, various degrees of obesity, and ectopic ossifications often localized in the subcutaneous tissue) (4). Not all traits can occur; they can also change in severity and evolve. Approximately 30% of patients presented symptoms constituting the main criteria for diagnosing AHO (brachydactyly and short stature). Moreover, the typical clinical features of PHP types and related disorders (PPHP, POH, acrodysostosis) make it difficult to make a precise diagnosis. For example, brachydactyly, typically occurring in acrodysostosis (92-97%) and PHP1A (79-80%), is less common in PHP1B (15-33%) and PPHP (<30%) (4). Moreover, brachydactyly is not specific to PHP and related disorders because it also occurs in Turner syndrome, tricho-rhino-phalangeal syndrome, deletion of chromosome 2q37, and as isolated brachydactyly type E (21). Therefore, genetic examination is important to verify the diagnosis, even in cases where the clinical picture suggests a specific disease entity. Knowing the exact molecular basis of the disease allows you to exclude or confirm PHP, which in turn will enable you to plan further care with the awareness of the possible discovery of further abnormalities in the future. According to the literature, familial PHP1B is mostly caused either by multiple exon deletions disrupting the STX16 gene or (less frequently) by deletions involving NESP. In our study, a family presenting PHPIB phenotype showed aberrant GNAS locus methylation and an intragenic deletion within the STX16 gene (22).

Additionally, identifying patients with these disorders is important for additional surveillance and subsequent studies on the incidence, natural history, and characterization of disease phenotypes. Ectopic ossifications are a direct clinical manifestation of molecular changes in the GNAS gene; they are not calcifications and do not depend on the concentration of calcium and phosphates in the blood. The essence of these changes is the appearance of additional foci of osteoblasts forming islands of ectopic bone tissue in the skin or the subcutaneous tissue. They can occur in diseases caused by reduced activity of subunits of G-stimulator protein (PHP1A, PHP1C, PPHP, and POH). They appear very rarely. The differential diagnosis should primarily consider fibrodysplasia ossificans progressiva (FOP) (15, 16).

The goal of PHP treatment is to reduce the early and late clinical symptoms of hypocalcemia and hyperphosphatemia. The doses of activated vitamin D metabolites (alfacalcidol or calcitriol) and oral calcium supplements (most often calcium carbonate) are determined individually, and their range is wide. Demand for medications may change over time, so it is important to monitor calcium and phosphate metabolism parameters periodically. In patients with normocalcemia, treatment with active vitamin D metabolites should be considered when the PTH concentration is twice the upper limit of normal (23).

As many as 84.2% ​​of our patients take L-thyroxine (25–112 mcg/day) (see **Table 4**), but determining the etiology of hypothyroidism is difficult in most cases. Due to chronic autoimmune thyroiditis commonly occurring in Poland, obtaining an increased concentration of antithyroid antibodies in laboratory tests does not exclude the co-occurrence of TSH resistance.

The analysis of the medical history of patients with PHP under the care of the Department of Endocrinology and Metabolic Diseases, PMMH-RI in Lodz allows us to confirm that the diagnosis of the disease is difficult due to the diversity of the clinical picture. To quickly establish the correct diagnosis, (1) it is crucial to collect detailed medical interviews with family history of the disease. (2) It is also essential to carefully perform a physical examination, looking for features of AHO or non-specific hypocalcemia symptoms, and (3) to check a broader panel of biochemical tests at the beginning of the diagnosis, characterizing calcium-phosphate homeostasis rather than just the concentration of total calcium in serum.

## Data Availability

The original contributions presented in the study are included in the article/supplementary material. Further inquiries can be directed to the corresponding author.
